# High‑flow nasal cannula is not more effective than conventional oxygen therapy for acute exacerbation of COPD with mild hypercapnia: we are not sure

**DOI:** 10.1186/s13054-022-04022-z

**Published:** 2022-05-31

**Authors:** Sergey N. Avdeev, Andrey I. Yaroshetskiy, Galia S. Nuralieva, Ivan S. Avdeev, Anna E. Shmidt

**Affiliations:** 1 Department of Pulmonology, I.M. Sechenov First Moscow State Medical University (Sechenov University), Healthcare Ministry of Russia, Trubetskaya Street 8, Moscow, Russia 119991; 2 Institute of Clinical Medicine, I.M. Sechenov First Moscow State Medical University (Sechenov University), Moscow, Russia

**To the Editor**

We read with great interest the article published in Critical Care by Xia et al. entitled “High-flow nasal cannula versus conventional oxygen therapy in acute COPD exacerbation with mild hypercapnia: a multicenter randomized controlled trial” [1]. In this randomized controlled trial, the authors aimed to compare conventional oxygen therapy (COT) and high-flow nasal cannula (HFNC) in hospitalized patients with acute exacerbations of chronic obstructive pulmonary disease (COPD) and mild hypercapnia (pH ≥ 7.35, PaCO_2_ > 45 mmHg). In this group of non-acidotic COPD patients with acute exacerbation, noninvasive ventilation does not facilitate recovery and, in addition, a substantial proportion of patients poorly tolerate noninvasive ventilatory support [2]. Therefore, we still need high-quality research on effective respiratory support in these patients. Xia et al. concluded that HFNC did not reduce the need for intubation and invasive mechanical ventilation in hospitalized patients with COPD exacerbation and mild hypercapnia; moreover, it increased the length of hospital stay and hospital costs. Nevertheless, some points require additional discussion.

First, we are not entirely sure about the presence of acute exacerbation in all enrolled patients with COPD. According to the data presented in the article, COPD patients had mild hypoxemia (median SpO_2_ 93%) and did not have tachypnea (median respiratory rate 21/min) and tachycardia (median heart rate 85–88/min), and also the levels of leukocytes and CRP were not elevated. Thus, it is highly likely that exacerbation of respiratory symptoms in COPD patients may not be exacerbations of COPD [3]. That is, worsening of respiratory symptoms in patients with COPD may also be caused by exacerbation of comorbidities (e.g., decompensated heart failure, arrhythmias, etc.), without involving the airways and lung (in these cases the term exacerbation of COPD may be misleading) [3]. And relatively good patient’s outcomes are also not in favor of an acute exacerbation of COPD: Among 330 enrolled patients, the intubation rate was 1.5% (5 patients) and in-hospital mortality was only 0.3% (1 patient). Also, the authors did not mention anything about the place of management of COPD patients; it is unlikely that the patients were managed in the ICU.

Second, the necessity for oxygen therapy in the enrolled patients is also questionable. Inclusion criteria for the study were pH and PaCO_2_ (but not PaO_2_). According to the data presented, the initial mean values of PaO_2_ were about 70 mmHg (it is not clear, while breathing room air or during O_2_ inhalation 2 L/min), that is, many patients simply did not require even conventional oxygen therapy. Also, Fig. 3 and Table E1 demonstrate that the respiratory rate, dyspnea, and blood gases remained practically unchanged during the first 3 days of oxygen therapy, that is, oxygen therapy was not accompanied by noticeable clinical effects.

Third, the oxygen therapy algorithm in this study also differs from accepted practice. The authors used SpO_2_ of 90–95% as target values, while randomized trials and observational studies have shown that in acute exacerbation of COPD, even with normocapnia, the most optimal target SpO_2_ level is 88–92% [4, 5]. Also, from Table E2 we can see that oxygen therapy was not continuous (about 10 h on the first day in the HFNC group and about 15 h a day in the COT group), which also differs from the usual practice.

These points raise some concerns, and we feel that the results of this study should be interpreted with caution and unlikely to affect our current practice. Hence, further research is required on the role of HFNC in non-acidotic acute exacerbation of COPD.

## Data Availability

Not applicable.

## Abbreviations

COPD: Chronic obstructive pulmonary disease
COT: Conventional oxygen therapy
FiO_2_: Fraction of inspiration oxygen
HFNC: High-flow nasal cannula
PaCO_2_: Arterial partial pressure of carbon dioxide
PaO_2_: Arterial partial pressure of oxygen
SpO_2_: Oxygen saturation
